# Prevalence of different stages of periodontal diseases among a sample of young adult obese Egyptian patients: a hospital based Cross-sectional study over 1 year

**DOI:** 10.1186/s12903-023-03278-3

**Published:** 2023-08-16

**Authors:** Yasmine Abbas, Basma Elsaadany, Noha Ghallab

**Affiliations:** 1 Periodontology Department, Faculty of Dentistry, Egyptian Russian University, Badr City, Egypt; 2 Oral Medicine & Periodontology, Faculty of Dentistry, Cairo University, Cairo, Egypt

**Keywords:** Obese, Prevalence, Periodontitis, AAP/EFP classification, Egyptian

## Abstract

**Background:**

This cross-sectional study aimed to determine the prevalence of different stages of periodontal diseases based on the recent classification in a sample of young adult obese Egyptian dental outpatients.

**Methods:**

This study included 314 patients seeking dental treatment at the Diagnostic Center of the Dental hospital, Faculty of Dentistry, Cairo University. Validated oral health questionnaire for adults regarding their age, gender, level of education and oral health routines as well as oral health impact profile questionnaire for chronic periodontitis (OHIP-CP) were filled by all patients. Obesity parameters were also assessed through person’s weight in kilograms, height in centimeters and waist circumference to determine the obesity stage. Diagnosis was made based on measurements of clinical periodontal parameters including a full mouth plaque index (PI), bleeding on probing (BoP), pocket depth (PD), clinical attachment level (CAL) and gingival recession depth (RD). Radiographic examination was performed using periapical radiographs. Ordinal logistic regression analysis was used to determine significant predictors of periodontal diseases and discriminant analysis was performed to predict periodontal disease classification.

**Results:**

The age range in the study sample was 19–39 years old. The prevalence of different stages of periodontal diseases was 100%. Gingivitis was the most prevalent periodontal disease (63.7%) followed by Periodontitis Stage III (22.6%) then Stage II (11.1%). Stage I showed the least prevalence (2.5%). An increase in BMI was statistically associated with an increase in PD, CAL, RD, PI and vice versa (*P*-value < 0.05). The total OHIP-CP was 15.99 ± 3.06 for all participants.

**Conclusions:**

There was a statistically significant association between periodontal diseases and obesity in young adults, as well as a statistically significant direct correlation between BMI and periodontal parameters. Self-assessment of oral health and obesity were significant predictors of periodontal disease.

**Clinical trial Registration ID:**

NCT04618068.

## Background

Obesity is one of the major health problems in the world, which increases the risk of several diseases including periodontitis [1]. Obesity-induced inflammation may enhance the susceptibility of the host to periodontal breakdown. Obesity and periodontitis are likely caused by an imbalance between the immunological and inflammatory systems of the body. Investigations are still ongoing on the biological mechanisms underlying the potential pathological link between periodontitis and obesity. The pathophysiology of obesity and periodontitis may be influenced by the same pathways since adipose tissue actively secretes a range of hormones and cytokines related to the inflammatory processes [2, 3].

Periodontitis and metabolic disorders including type 2 diabetes and obesity are associated with a bidirectional relationship [4] Numerous biological factors of obesity were linked to the pathogenesis of periodontitis owing to the changes in the pro- and anti-inflammatory cytokines resulting in a state of hyper-inflammatory host response [5]. Systemic low-grade inflammation in turn stimulates host responses and promotes the release of inflammatory cytokines like tumor necrosis factor-alpha (TNF-α), interleukins (IL) as IL-1 beta and IL-6, which is likely the common factor between these chronic conditions [6].

A systematic review conducted by Moura-Grec et al. [7] concluded that obese people have a significantly higher risk of developing periodontal diseases compared to non-obese people*.* Recently, Da Silva et al. [8], conducted another systematic review and meta-analysis and studied the link between gingival inflammation and obesity. The authors demonstrated that obese patients with periodontitis have significantly greater levels of gingival inflammation.

Currently, there is scarce data in the periodontal literature concerning periodontal disease prevalence among obese subjects in the Arab African population. Hence, there is a need for epidemiological studies representing the adult population from this region to enable proper development of guidelines and appropriate public health programs [9, 10]. Furthermore, most epidemiologic studies used a wide range of case definitions which had an intense impact on the reported periodontal disease prevalence in several populations. This called for the use of a standard definition for periodontal disease in epidemiologic studies [11]. Accordingly, this cross-sectional study applied the new EFP/AAP classification of periodontal disease in diagnosing patients based on the recently introduced staging and grading system [12–14]. Although a recent cross-sectional study investigated the frequency and risk indicators/predictors of periodontitis in a sample of Egyptian adult population, yet obesity was not addressed [15]. Based on the above-mentioned findings, the research question of this current cross-sectional study was “what is the prevalence of periodontal diseases according to the new classification system among a sample of young adult obese Egyptian patients attending the diagnostic centre at the Faculty of Dentistry, Cairo University?”. To the best of the author’s knowledge, this is the first cross-sectional study examining the prevalence of periodontal disease based on the new EFP/AAP classification system among a sample of young adult obese Egyptian patients.

## Methods

### Study settings

The present observational cross-sectional study included 314 participants of young adult obese Egyptian patients seeking dental treatment at the Diagnostic centre of the Dental hospital, Faculty of Dentistry, Cairo University. Subjects were recruited in a consecutive manner from March 2021 till September 2021 and screening of patients was continued until the target sample was achieved.

### Ethical procedure

The study protocol was approved by the Research Ethics committee, Faculty of Dentistry, Cairo University [approval NO. 14 10 20]. The detailed steps were clearly described to all patients selected in this study. The aim of the study was explained to all subjects participating in this study and informed consents were obtained before filling the questionnaire.

### Eligibility and exclusion criteria

#### Inclusion criteria

This study included patients whose age was between 18–39 years, body mass index more than 30 kg/m^2^ and approved to provide informed consent.

#### Exclusion criteria

The following patients were excluded; patients with any systemic diseases or on medication for systemic disease/conditions, completely edentulous patients, patients having removable prosthetic appliance, patients who received periodontal treatment or antibiotics for at least 3 months, pregnant women or lactating mothers and patients having problem in opening their mouth or undergoing intermaxillary fixation.

### Power and sample size calculation

The present power analysis used effect of obesity on periodontal health as the primary outcome. With a margin of error 5%, expected frequency of 71.3% [16], at confidence level of 95%, the minimum estimated sample size was 314 subjects. Sample size calculation was performed using Epi Info 7.2.2.2.

### Addressing potential sources of bias

#### Non-respondent bias

Patients refusing to participate (non-respondents) were asked about the cause of their refusal and received explanation of their value to the study even if they were not complaining of any oral lesions.

#### Selection bias

Patients were included in the study through consecutive sequence of entry to the diagnostic center.

### Interview & data collection

#### Oral health questionnaire for adults

A structured questionnaire was completed for each patient by a single examiner (AEY). This oral health questionnaire for adults was selected according to the world health organization (WHO) [17]. It included several questions about the sociodemographic data, oral hygiene habits, frequency of dental visits and smoking. All the questions were explained and translated into Arabic language. The secondary outcome of the study was the Oral Health Impact Profile questionnaire for Chronic Periodontitis (OHIP-CP) [18].

### Clinical periodontal & radiographic examination

Clinical and radiographic examinations were performed by a trained examiner (AEY) to reach a case identification and diagnose the periodontal condition of the participants according to the latest EFP/AAP classification of periodontal diseases [13, 14]. Based on the clinical and radiographic assessment, the recruited participants were either diagnosed as gingivitis or periodontitis and the stages of periodontitis were also identified. Alveolar bone loss was determined by taking digital periapical radiographs for the most affected area.

### Clinical periodontal parameters

Diagnosis and case identification of the periodontal disease was performed based on the new classification of periodontal disease [14]. To diagnose and define the stage of periodontitis, the following periodontal parameters were recorded for all participants; plaque index (PI) [19], dichotomous bleeding on probing (BoP) expressed as percentage [20], pocket depth (PD), clinical attachment level (CAL) and gingival recession depth (RD). Gingival bleeding was assessed by gentle probing of the orifice of the gingival crevice. The periodontal probe was inserted 1 to 2 mm into the gingival sulcus starting at one interproximal area and moving to the other. If bleeding occurs within 10 s a positive finding was recorded. PD was measured from the free-gingival margin to the base of the pocket with a light force applied, CAL was measured from the cemento-enamel junction (CEJ) to the base of the pocket and RD was measured from the CEJ to the most apical extension of the gingival margin [21]. Measurements were recorded at six sites for all teeth mesio-buccal, mesio-lingual, mid-buccal, disto- buccal, disto-lingual, and mid-lingual using William’s graduated periodontal probe (Martin™ graduated periodontal probe No. 43–357-00, KLS Martin Group, Germany) and were rounded to the highest whole millimetre.

### Obesity parameters

Person’s weight in kilograms: was recorded using a standard physician’s scale.

Height in centimetres: was taken using a stadiometer.

Waist circumference (WC): was measured using measuring tape at the midpoint between the lower border of ribs and upper border of pelvis.

Body mass index (BMI): According to WHO expert report BMI was calculated by dividing the weight by the square of his/her height in meters (kg/m^2^) [22]. Obesity was classified according to BMI scores as class I obesity 30–34.9, class II obesity 35–39.9 and class III obesity ≥ 40.

### Statistical analysis

The current statistical methods were guided by Zokari et al. [23]. Frequencies and percentages were used to present qualitative data. The mean, standard deviation (SD), and 95% Confidence Interval (CI) for quantitative data were displayed together with the median and range values. In univariate analysis, comparisons of qualitative variables were made using the Chi-square test or Fisher's Exact test. Quantitative data were reviewed for normality by checking the distribution of data and using tests of normality (Kolmogorov–Smirnov and Shapiro–Wilk tests). For parametric data, Student’s t-test was used to compare between patients with Class I and Class II Obesity. For non-parametric data, Mann–Whitney U test was used for the comparisons. Spearman’s correlation coefficient was used to determine the correlation between different quantitative data. For multivariate analysis; ordinal logistic regression analysis was used to determine significant predictors of periodontal diseases. Model fit was tested using Chi-square test and Pseudo R2 tests and the model was fit to describe the relations between the dependent and independent variables. The regression coefficient (b), standard error (SE), and 95% CI were calculated. Discriminant analysis was performed to determine discriminant functions for predicting periodontal disease classification based upon data of age, gender, BMI, waist circumference, self-assessment of oral health, obesity class, PD, CAL, RD, BoP and PI. The significance level was set at *P* ≤ 0.05. Statistical analysis was performed with IBM SPSS Statistics for Windows, Version 23.0. Armonk, NY: IBM Corp.

## Results

### Demographic data and basic characteristics of the study participants

Demographic data of the study participants are shown in Table 1.

**Table 1:** Descriptive statistics for demographic data and basic characteristics of the study participants (*n* = 314)

**Demographic data**	
**Gender [n (%)]**	
Male	141 (44.9%)
Female	173 (55.1%)
**Age (Mean ± SD)**	32.2 ± 4.8
**Residence n (%)**	
Urban	230 (73.2%)
Rural	84 (26.8%)
**Education [n (%)]**	
Less than primary school	3 (1%)
Primary school completed	4 (1.3%)
Secondary school completed	18 (5.7%)
High school completed	43 (13.7%)
College/University completed	236 (75.2%)
Postgraduate degree	10 (3.2%)
**Smoking habits**	
Yes	125 (39.8%)
No	189 (60.2%)
**The prevalence of alcohol drinking in last 30 days**	
Yes	4 (1.3%)
No	189 (60.2%)
**Self-assessment of oral health**	
Excellent	3 (1%)
very good	93 (29.6%)
good	120 (38.2%)
average	76 (24.2%)
poor	17 (5.4%)
very poor	5 (1.6%)
**Frequency of cleaning teeth**	
Never	6 (1.9%)
Once a month	11(3.5%)
2–3 times a month	42(13.4%)
Once a week	95(30.3%)
2–6 times a week	100(31.8%)
Once a day	48(15.3%)
Twice or more a day	12(3.8%)
**Method of cleaning**	
Tooth brush	307/308(99.7%)
Wooden tooth picks	17/308(5.5%)
Plastic tooth picks	7/308(2.3%)
Dental floss	13/308(4.2%)
Mouthwash	31/308(10.1%
Miswak\chew stick	10/308(3.2%)
**Use of tooth paste**	
Yes	308/308(100%
No	0/308 (0%)

### Oral Health Impacts Profile (OHIP-CP) questionnaire

Distribution of the responses to OHIP-CP questionnaire are presented in Table 2. The mean ± SD values for all participants of the total OHIP-CP was 15.99 ± 3.06 with a median value of 16 and the range was 10 – 31.

**Table 2:** Frequencies (n) and percentages (%) for OHIP-CP questionnaire (*n* = 314)

OHIP-CP dimensions	Never	Hardly ever	Occasionally	Fairly often	Very often
**Pain and Functional limitation**					
1. Have you had toothache because of problems with your teeth or mouth?	0 (0)	0 (0)	314 (100)	0 (0)	0 (0)
2. Have you had painful gums because of problems with your teeth or mouth?	0 (0)	0 (0)	313 (99.7)	1 (0.3)	0 (0)
3. Have you had sensitive teeth, due to hot or cold foods or drinks, because of problems with teeth or mouth?	89 (28.3)	61 (19.4)	56 (17.8)	66 (21)	42 (13.4)
4. Have you ever had any teeth become loose on their own, without an injury?	314 (100)	0 (0)	0 (0)	0 (0)	0 (0)
5. Have you had difficulty chewing any foods because of problems with your teeth or mouth?	98 (31.2)	103 (32.8)	101 (32.2)	12 (3.8)	0 (0)
6. Have you ever had bleeding gums (spontaneous bleeding or while brushing your teeth or biting hard objects?	0 (0)	0 (0)	205 (65.3)	109 (34.7)	0 (0)
7. Have you felt your breath has been stale because of problems with your teeth or mouth?	0 (0)	0 (0)	313 (99.7)	1 (0.3)	0 (0)
8. Have you had food catching in your teeth?	0 (0)	0 (0)	312 (99.4)	2 (0.6)	0 (0)
**Psychological discomfort**					
9. Have you felt your sense of taste has worsened because of problems with your teeth or mouth?	311 (99)	2 (0.6)	1 (0.3)	0 (0)	0 (0)
10. Have you been self-conscious because of your teeth or mouth?	34 (10.8)	259 (82.5)	15 (4.8)	6 (1.9)	0 (0)
11. Have you felt tense because of problems with your teeth or mouth?	33 (10.5)	261 (83.1)	16 (5.1)	4 (1.3)	0 (0)
12. Have you avoided smiling (i.e., noticed that gaps had developed between your front teeth or that you had swollen or abscess gums) because of problems with your teeth or mouth?	279 (88.9)	1 (0.3)	29 (9.2)	5 (1.6)	0 (0)
13. Have you been embarrassed because of your teeth or mouth?	290 (92.4)	0 (0)	20 (6.4)	4 (1.3)	0 (0)
14. Have you avoided going out because of your teeth or mouth?	293 (93.3)	2 (0.6)	17 (5.4)	2 (0.6)	0 (0)
**Psychological disability and social handicap**					
15. Have you been unable to brush teeth because of your teeth or mouth?	277 (88.2)	2 (0.6)	25 (8)	9 (2.9)	1 (0.3)
16. Has your diet been unsatisfactory because of problems with your teeth or mouth?	305 (97.1)	1 (0.3)	6 (1.9)	2 (0.6)	0 (0)
17. Have you felt your general health has worsened because of problems with your teeth or mouth?	314 (100)	0 (0)	0 (0)	0 (0)	0 (0)
18. Have you felt that life in general was less satisfying because of problems with your teeth or mouth?	313 (99.7)	1 (0.3)	0 (0)	0 (0)	0 (0)

### Prevalence of different stages of periodontal diseases

Prevalence of different stages of periodontal diseases in the study sample was as following: 100% has periodontal disease (63.8% gingivitis, 2.5% stage I periodontitis, 11.1% stage II periodontitis and 22.6% stage III periodontitis).

### Periodontal examination

Descriptive statistics for PD, CAL, RD, BoP and PI are presented in Table 3.

**Table 3:** Descriptive statistics for pocket depth (PD), clinical attachment level (CAL), gingival recession depth (RD), bleeding on probing (BoP) and plaque index (PI)

Periodontal disease	Periodontal parameters	Mean	SD	95% CI	Median	Minimum	Maximum
**Gingivitis (*n* = 200)**	PD (mm)	2.3	0.2	2.27–2.33	2.3	1.7	2.9
	CAL (mm)	0	0	0–0	0	0	0
	RD (mm)	0	0	0–0	0	0	0
	BOP (%)	97.33	5.39	96.57–98.08	100	66	100
	PI (Score)	1.82	0.52	1.75–1.89	2	0.2	3
**Periodontitis Stage I (*n* = 8)**	PD (mm)	2.43	0.23	2.24–2.61	2.35	2.2	2.9
	CAL (mm)	1.75	0.46	1.36–2.14	2	1	2
	RD (mm)	0.75	1.04	-0.12–1.62	0	0	2
	BOP (%)	96.88	5.84	91.99–101.76	100	86	100
	PI (Score)	2.36	0.34	2.08–2.65	2.35	2	3
**Periodontitis Stage II (*n* = 35)**	PD (mm)	2.38	0.28	2.29–2.47	2.3	1.8	3.1
	CAL (mm)	3.77	0.43	3.63–3.92	4	3	4
	RD (mm)	0.53	0.79	0.26–0.8	0	0	2
	BOP (%)	97.11	6.23	94.97–99.26	100	71	100
	PI (Score)	2.02	0.57	1.82–2.21	2	1	3
**Periodontitis Stage III (*n* = 71)**	PD (mm)	2.51	0.24	2.45–2.56	2.5	2.1	3.3
	CAL (mm)	5.2	0.47	5.09–5.31	5	5	7
	RD (mm)	0.83	1.11	0.57–1.09	0	0	3
	BOP (%)	95.51	7.84	93.65–97.36	100	61	100
	PI (Score)	2.4	0.45	2.29–2.51	2.4	1.4	3

### Body Mass Index (BMI) and obesity

The mean (± SD) body weight was 109.6 (± 8.1) Kg, height was 167.2 (± 5.5) cm, waist circumference was 115.2 (± 8.5) cm and BMI was 32.9 (± 1.9) (Kg/m^2^). Most participants had class I obesity (85%), while class II obesity patients represented 15% of the study participants.

### Univariate analysis

#### Association between obesity and demographic data

There was no statistically significant association between gender, residence, education and obesity (*P*-value = 0.06, 0.38 and 0.44, Effect size = 0.60, 1.28 and 0.12 respectively). Patients with obesity class I had a mean ± SD age of 31.9 ± 4.9 years, compared to 33.6 ± 4.4 years for those with obesity class II. Current statistical analysis showed that patients with obesity class I showed significantly lower mean age than patients with obesity Class II (*P*-value = 0.030, Effect size = 0.345).

### Associations between obesity, and oral health behavior, oral health self-assessment, smoking, drinking habits periodontal disease stage

There was a statistically significant association between obesity and different stages of periodontal diseases (*P*-value = 0.006, Effect size = 0.199). Patients with obesity class I showed higher prevalence of gingivitis and lower prevalence of all stages of periodontitis compared to patients with obesity class II. However, there was no statistically significant association between obesity and oral health behaviors as well as smoking and drinking habits as shown in Table 4.

**Table 4:** Descriptive statistics and results of Fisher’s Exact test for comparison between oral health behavior, oral health self-assessment, smoking, drinking habits and periodontal disease stage among obese patients

	**Obesity class I (*n* = 267)**	**Obesity class II (*n* = 47)**	***P*-value**	**Effect size (v)**
**Frequency of cleaning teeth**				
Never	6 (2.2)	0 (0)	0.275	0.157
Once a month	8 (3)	3 (6.4)		
2–3 times a month	31 (11.6)	11 (23.4)		
Once a week	82 (30.7)	13 (27.7)		
2–6 times a week	87 (32.6)	13 27.7)		
Once a day	43 (16.1)	5 (10.6)		
Twice or more a day	10 (3.7)	2 (4.3)		
**Method of cleaning teeth**				
Tooth brush	260 (99.6)	47 (100)	1	0.847
Wooden tooth picks	16 (6.1)	1 (2.1)	0.486	1.118
Plastic tooth picks	6 (2.3)	1 (2.1)	1	1.012
Dental floss	12 (4.6)	1 (2.1)	0.700	1.094
mouthwash	29 (11.1)	2 (4.3)	0.193	1.117
Miswak\chew stick	8 (3.1)	1 (2.1)	1	1.051
**Self-assessment of oral health**				
Excellent	3 (1.1)	0 (0)	0.406	0.133
Very good	82 (30.7)	11 (23.4)		
Good	104 (39)	16 (34)		
Average	60 (22.5)	16 (34)		
Poor	13 (4.9)	4 (8.5)		
Very poor	5 (1.9)	0 (0)		
**Smoking**				
Never	165 (61.8)	24 (51.1)	0.422	0.084
Once a week	1 (0.4)	0 (0)		
Several times a week	14 (5.2)	3 (6.4)		
Everyday	87 (32.6)	20 (42.6)		
**Drinking alcohol**				
1 drink	1 (0.4)	0 (0)	1	0.048
2 drinks	2 (0.7)	0 (0)		
3 drinks	1 (0.4)	0 (0)		
Non-drinker	263 (98.5)	47 (100)		
**Periodontal diseases**				
Gingivitis	180 (67.4)	20 (42.6)	0.006*	0.199
Periodontitis Stage I	6 (2.2)	2 (4.3)		
Periodontitis Stage II	29 (10.9)	6 (12.8)		
Periodontitis Stage III	52 (19.5)	19 (40.4)		

^*^Significant at *P* ≤ 0.05

### Association between obesity and periodontal parameters in patients with different periodontal diseases

There was no statistically significant difference between patients with class I and class II obesity regarding CAL, RD and BoP in different periodontal diseases. However, in patients with periodontitis stage I, obesity class I patients showed a statistically significant lower mean PD than patients with obesity Class II. Moreover, in patients with periodontitis stage III, obesity class I patients showed a statistically significant lower mean PI than patients with obesity Class II Table 5.

**Table 5:** Mean ± SD and results of Student’s t-test and Mann–Whitney U test for comparison between periodontal parameters in patients with Class I and Class II Obesity at different stages of periodontal diseases

Periodontal parameters	Obesity Class I	Obesity Class II	*P*-value	*Effect size (d)*
**Gingivitis**				
PD (mm)	2.3 ± 0.2	2.34 ± 0.24	0.389	0.204
CAL (mm)	0.0 ± 0.0	0.0 ± 0.0	- -	
RD (mm)	0.0 ± 0.0	0.0 ± 0.0	1	0
BOP (%)	97.21 ± 5.59	98.4** ± **2.87	0.348	0.222
PI (Score)	1.82 ± 0.51	1.86 ± 0.62	0.735	0.048
**Periodontitis stage I**				
PD (mm)	2.32 ± 0.08	2.75 ± 0.21	0.003*	3.92
CAL (mm)	1.83 ± 0.41	1.5** ± **0.71	0.420	0.707
RD (mm)	0.67** ± **1.03	1** ± **1.41	0.693	0.237
BOP (%)	95.83** ± **6.52	100** ± **0	0.424	0.7
PI (Score)	2.43** ± **0.36	2.15** ± **0.21	0.241	0.906
**Periodontitis stage II**				
PD (mm)	2.39 ± 0.29	2.32** ± **0.17	0.545	0.274
CAL (mm)	3.72 ± 0.45	4** ± **0	0.152	0.658
RD (mm)	0.53 ± 0.8	0.5** ± **0.84	0.938	0.022
BOP (%)	96.76** ± **6.71	98.83** ± **2.86	0.466	0.331
PI (Score)	2** ± **0.57	2.1** ± **0.61	0.759	0.104
**Periodontitis stage III**				
PD (mm)	2.49 ± 0.23	2.54** ± **0.27	0.509	0.178
CAL (mm)	5.19 ± 0.44	5.21** ± **0.54	0.885	0.039
RD (mm)	0.71 ± 1.05	1.16** ± **1.21	0.139	0.31
BOP (%)	94.98** ± **8.58	96.95** ± **5.21	0.353	0.251
PI (Score)	2.32** ± **0.44	2.64** ± **0.4	0.014*	0.6

^*^Significant at *P* ≤ 0.05

### Correlation between BMI and periodontal parameters

The present statistical analysis revealed a significant direct correlation between BMI and periodontal parameters (*P*-value < 0.05) including; PD, CAL, RD and PI. Correlation coefficient (ρ) was (0.11, 0.24, 0.16 and 0.193 respectively). This denotes that an increase in BMI was associated with an increase in PD, CAL, RD, PI and vice versa.

### Multivariate analysis

#### Ordinal regression analysis

Ordinal regression model was constructed using periodontal disease (ordinal variable on the following scale: Gingivitis = 1, periodontitis Stage I = 2, periodontitis Stage II = 3 and periodontitis Stage III = 4) as the dependent variable. Dental history, self-assessment of oral health, frequency of cleaning teeth, time since last dental visit, smoking, drinking alcohol, OHIP-CP and obesity were the independent variables. The model was adjusted for the following covariates: gender, age, educational level and residence. Results of the regression model showed the following statistically significant predictors Table 6. Self-assessment of oral health was found to be a statistically significant predictor of periodontal disease. Subjects with very poor, poor and average oral health condition were associated with higher grades of periodontal diseases. Obesity was found to be a statistically significant predictor of periodontal disease. Subjects with Class I Obesity were associates with lower grades of periodontal diseases.

**Table 6:** Results of ordinal regression analysis model showing significant predictors of periodontal disease

Variable	Regression coefficient (b)	Standard Error (SE)	*P*-value	95% CI
**Self-assessment of oral health**				
Very poor	18.265	0.85	< 0.001*	16.6–19.93
Poor	16.82	0.453	< 0.001*	15.931–17.708
Average	14.881	0.413	< 0.001*	14.073–15.69
**Obesity (Class I)**	-0.934	0.369	0.011*	-1.656- -0.211

^*^Significant at *P* ≤ 0.05

### Discriminant analysis and equations for classifying periodontal disease among Egyptian population Using age, gender, BMI, waist circumference, PD, CAL, RD, BOP and PI as predictors

The analysis resulted in the following equation:

D = 0.111 Gender – 0.001 Age + 0.013 BMI -0.004 WC -0.028 PD + 2.936 CAL -0.131 RD – 0.011 BOP -0.176 PI – 3.868.

The discriminate functions at group centroids (Group means) were -5.277, 0.427, 7.487 and 11.126 for gingivitis, periodontitis Stage I, Stage II and Stage III, respectively. This indicates that a patient with a score close to any of the previous scores will be classified as having the corresponding stage of periodontal disease. Classification results revealed that 100% of gingivitis patients, 100% of periodontitis Stage I patients, 91.4% of Stage II patients and 88.7% of Stage III patients were correctly classified according to the previous discriminant function. The overall correct classification was 96.5%.

## Discussion

This hospital-based cross-sectional study aimed at determining the prevalence of periodontal diseases implementing the new classification of periodontal disease (12–14) among a sample of young adult obese Egyptian patients extracted from the outpatients’ clinic at Faculty of Dentistry, Cairo University. The present population sample was believed to be an appropriate representative for the target adult population in Egypt being one of the largest hospitals in Egypt with a population of 223,200 patients/year. Moreover, most of the cross-sectional studies investigating prevalence of periodontal disease were hospital-based rather than population-based studies due to the frequency of consultations at hospital settings [24].

Obesity is a chronic, multifactorial disease that leads to an excessive deposition of fat in the adipose tissue [25]. Numerous prevalent health conditions, including diabetes, cardiovascular disease, hypertension, osteoarthritis and periodontitis are significantly affected by obesity [26]. Previous research has shown that obesity raises the systemic inflammatory burden owing to the increased immunological and metabolic factors, which enhances periodontal disease susceptibility [27]. A positive correlation between obesity and periodontal disease have been reported according to a recent systematic review [28]. In addition, it has been hypothesized that obesity is among the greatest risk factors for the inflammatory destruction of periodontal tissue after smoking [29].

The pathways through which obesity may contribute to an increase in periodontal disease have been the focus of current research. The link between obesity and periodontitis is most likely due to the same inflammatory process connecting the two conditions [30]. Individuals who are obese frequently have an aggravated inflammatory response. Because of alterations in adipose tissues, obesity is regarded as a chronic low-grade inflammatory disease [31]. Even though numerous investigations studied the prevalence of periodontal diseases among obese patients in different communities around the world [29, 32–34], there is scarce data available for the Egyptian population. Currently, there is inconclusive evidence about the relationship between the severity of periodontitis in terms of different stages of periodontal diseases and young adult obese patients [32]. Consequently, the goal of the current cross-sectional study was to assess the prevalence of different stages of periodontal diseases based on the new EFP/AAP classification and in young adults with obesity.

Definition of periodontal disease in the present study was according to the most recent classification of periodontal disease adopted by the EFP/AAP. Cases were identified, diagnosed, and staged using clinical periodontal parameters [35]. The advantage of this classification over others is that it provides information on the severity, diagnosis, pathogenesis and necessary treatments for periodontal diseases [13, 14].

Collectively, the current observations revealed that 100% of the participants showed periodontal diseases and 35.3% suffered from different stages of periodontitis. It is worthy to mention that this cross-sectional study was conducted on a sample of young adult obese Egyptian patients attending the diagnostic center at the faculty of dentistry, Cairo University. These findings were supported by a recent cross-sectional study investigating the prevalence of periodontal disease among 750 Egyptian patients recruited from the same hospital [15]. The later study concluded that gingivitis was the most frequent periodontal disease (39.6%) followed by periodontitis stage I (38%), stage II (20.4%), stage III (1.6%) and stage IV (0.4%). Egyptian patients attending this public hospital mainly seek treatment only for pain along with having poor oral hygiene which explains why 100% of the participants showed periodontal diseases in the current study. These observations offer a major contribution to the periodontal health information of the Egyptian population encouraging raising awareness and providing preventive measures which could be beneficial for policy makers to provide comprehensive screening programs for the assessment of oral health and periodontal treatment needs for obese patients at an early age.

The current statistical analysis showed a significant direct correlation between BMI and periodontal parameters including PD, CAL, RD and PI. Furthermore, there was a statistically significant association between periodontal diseases and obesity. Moreover, patients with obesity class I showed higher prevalence of gingivitis and lower prevalence of all stages of periodontitis than patients with obesity class II. Several studies reported a positive correlation/association between obesity and periodontal diseases including both obese and non-obese patients in different populations including; Taiwan [36], Japan [33, 34], Jordan [37], Egypt [38] and India [39]. Nevertheless, few cross-section studies were conducted on only obese patients as done in the present study to estimate the prevalence of periodontal diseases. The discrepancy was in the previous methods of defining periodontitis and the characteristics of the included patients’ such as age, medical status and degree of obesity [16, 40–42]. Based on the authors knowledge, the present study is the first cross-sectional study using the new EFP/AAP classification of periodontal disease characterized by a multidimensional staging system, to represent the relationship between different classes of obesity and the severity of periodontal disease.

In accordance with the results of present study, Colak et al. [42] estimated the prevalence of periodontitis according to the new classification but only in 30 patients with BMI greater than 40 undergoing bariatric surgery. The authors grouped all the periodontitis stages into one periodontitis group and gingivitis or healthy patients were considered as a non-periodontitis group. They found that the prevalence of periodontitis was 67%, which was almost double the prevalence presented in this study and reported that the increase in BMI was correlated to an increase in the periodontal parameters. These superior findings may be explained by the fact the authors included only old patients (mean age of 48.3 years) with BMI greater than 40 kg/m^2^.

In a Malaysian study, Khan et al. [40] reported that the prevalence of chronic periodontitis among an obese Malaysian population was 74%. They defined periodontitis by population-based surveillances of chronic periodontitis and included both young and old age adults. In contrast to the present findings, BMI was not correlated with periodontitis. In another cross-section study, Thomas et al. [16] defined periodontitis based on the chronic periodontitis index (CPI) and demonstrated a prevalence of 71.3% for periodontal disease in a sample of young obese Saudi patients. The authors found a significant association between BMI and loss of attachment among the 21–30-year-old study subjects, which was in accordance with the findings presented herein.

Moreover, another study investigating adult obese patients in Bahrain showed that the prevalence of periodontitis was 97% [41]. The high prevalence of periodontitis observed in the Bahraini obese participants might be due to the shortcomings of CPI that was used to define periodontitis. Therefore, the possibility to overestimate the prevalence cannot be excluded. In contrast to the currently presented finding, the authors reported that BMI was not correlated with periodontitis, yet the WC had a weak positive correlation.

The high prevalence of periodontal affection in obese subjects with a young age was previously reported [33, 40]. Al-Zahrani et al. [33] reported that BMI & WC were associated with prevalence of periodontal disease in individuals aged between 18 to 34 years old, but not in the middle age (35 to 59 years old) and old age groups (60 to 90 years old). While Khan et al. [40] found that among an obese Malaysian population chronic periodontitis was more prevalent in the age group of 30–39 years old.

It might be speculated that the high prevalence of periodontitis among the young age group could be related to the fact that this they belong to the working age group. Young individuals could be preoccupied with their daily routine; thus, they might not have time to visit the dental clinic regularly or care about oral hygiene. The busy lifestyle among the younge adults also could have indirectly induced stress which may have contributed to the overall burden of inflammation [43].

Periodontal disease is a public health problem, hence it is crucial to understand that well-structured oral health education programs based on nationally representative data would help in reducing prevalence of periodontitis among young obese individuals and raise awareness through training programs [44]. Policy makers are urged by data from cross-sectional studies to prioritize preventive and therapeutic public dental care facilities especially for obese subjects at an early age [45]. This cross-sectional study is a reflective of the Egyptian population and could pave the way for future research to investigate the prevalence of periodontal diseases among young obese individuals in the whole Egyptian population.

Nevertheless, the limitations of this investigation include; (i) being a hospital-based study limits the data' generalizability, (ii) being a cross-sectional study, it is not feasible to determine if obesity and periodontal disease are causally related and (iii) despite the sample size was calculated and the study power was accepted yet the sample size limited our ability for evaluating the relationship of obesity and periodontal diseases across subgroups (gender, educational level, smoking and alcohol intake). Therefore, longitudinal long-term studies with larger sample sizes are required to further understand the pathophysiology between periodontal disease and obesity.

Conclusion: In a sample of obese young adult Egyptian population, the prevalence of periodontal disease according to the recent AAP/EFP classification of periodontal diseases was 100%. Gingivitis was the most prevalent periodontal disease with a prevalence of 63.7%, while least prevalence 2.5% was seen in Stage I. In addition, there was a statistically significant direct correlation between BMI and periodontal parameters. Multivariate analysis revealed that self-assessment of oral health and obesity were significant predictors of periodontal disease.

## Data Availability

The datasets used and/or analyses used during the current study are available from the corresponding author upon reasonable request.

## Abbreviations

PD: Probing depth
CAL: Clinical attachment loss
RD: Recession depth
BoP: Bleeding on probing
PI: Plaque index
BMI: Body mass index
WC: Waist circumference
EFP/AAP: (European Federation of Periodontology/American Academy of Periodontology)
OHIP: Oral Health Impacts Profile
CPI: Chronic periodontitis index
